# Economic Conditions May Contribute to Increased Violence toward Children: A Nationwide Population-Based Analysis of Pediatric Injuries in Taiwanese Emergency Departments

**DOI:** 10.3390/ijerph15020182

**Published:** 2018-01-23

**Authors:** Yueh-Ping Liu, Ren-Jun Hsu, Mei-Hwan Wu, Chun-Chih Peng, Shu-Ting Chang, Wei-Te Lei, Tzu-Lin Yeh, Jui-Ming Liu, Chien-Yu Lin

**Affiliations:** 1 Department of Emergency Medicine, National Taiwan University Hospital, Taipei 100, Taiwan; dtemer14@gmail.com; 2 Department of Medical Affairs, Ministry of Health and Welfair, Taipei 115, Taiwan; 3 Graduate Institute of Life Sciences, National Defense Medical Center, Taipei 114, Taiwan; hsurnai@gmail.com; 4 Biobank Management Center of the Tri-Service General Hospital, National Defense Medical Center, Taipei 114, Taiwan; 5 Department of Pathology and Graduate Institute of Pathology and Parasitology, The Tri-Service General Hospital, National Defense Medical Center, Taipei 114, Taiwan; 6 Department of Pediatrics, National Taiwan University Hospital and College of Medicine, National Taiwan University, Taipei 100, Taiwan; wumh@ntu.edu.tw; 7 Department of Pediatrics, MacKay Children’s Hospital, Taipei 104, Taiwan; pengcc4566@gmail.com; 8 Department of Medicine, MacKay Medical College, New Taipei City 252, Taiwan; 9 Department of Pediatrics, BinKun Women’s and Children’s Hospital, Taoyuan 324, Taiwan; tototinforever@yahoo.com.tw; 10 Department of Pediatrics, Hsinchu MacKay Memorial Hospital, Hsinchu 300, Taiwan; lazyleisure@gmail.com (W.-T.L.); 5767@mmh.org.tw (T.-L.Y.); 11 Division of Urology, Department of Surgery, Taoyuan General Hospital, Ministry of Health and Welfare, Taoyuan 330, Taiwan; 12 Department of Medicine, National Yang-Ming University, Taipei 112, Taiwan

**Keywords:** pediatric injuries, unintentional injuries, National Health Insurance Research Database, NHIRD, emergency department, financial crisis, public health, economics

## Abstract

Childhood injuries are unfortunately common. Analysis procedures may assist professionals who work with children with developing preventive measures for protecting children’s wellness. This study explores the causes of pediatric injuries presenting to an emergency department in Taiwan. This nationwide, population-based study was conducted using data from the National Health Insurance Research Database of Taiwan (NHIRD). Patients aged <18 years were identified from approximately one million individuals listed in the NHIRD. We followed up with these patients for nine years and analyzed the causes of injuries requiring presentation to an emergency department. Variables of interest were age, sex, injury mechanisms, and temporal trends. A total of 274,028 children were identified in our study. Between 2001 and 2009, the leading causes of pediatric injuries treated in emergency departments were motor vehicle injuries, falls, and homicide. The overall incidence of injuries declined over the course of the study because of reductions in motor vehicle accidents and falls. The incidence of homicide increased during the study period, particularly between 2007 and 2009. A moderately inverse correlation between homicide rate and economic growth was observed (correlation coefficient: −0.613, *p* = 0.041). There was a general decline in pediatric injuries between 2001 and 2009. Public policy changes, including motorcycle helmet laws and increases in alcohol taxes, may have contributed to this decline. Unfortunately, the incidence of homicide increased over the course of the study. Ongoing financial crises may have contributed to this increase. Multidisciplinary efforts are required to reduce homicide and reinforce the importance of measures that protect children against violence.

## 1. Introduction

Children are susceptible to a variety of injuries and often seek medical help from caregivers in emergency departments [1]. Pediatric injuries are an important public health issue that exert a worldwide burden. Injuries are the leading cause of mortality in children, also contributing to morbidity [2,3,4,5]. According to Taiwan's official Vital Statistics System, injuries are the number one cause of mortality in children older than one year of age [6]. Pediatric injuries are classified into three groups based on intent: intentional, unintentional, and undetermined injuries. Globally, unintentional injuries were responsible for 15.4% of 2.6 million deaths among children aged one to 14 years in 2013 [2]. In the United States, unintentional home injuries account for more than 30,000 deaths, 12 million nonfatal injuries, and an estimated $222 billion in medical costs each year [7]. The impact of pediatric injuries is huge and threat of pediatric injuries warrants our attention.

Prevention is central to decrease the incidence, morbidity, and mortality of pediatric injuries. Understanding the spectrum of pediatric injuries facilitates preventive actions. Motor vehicle injuries (MVIs) are the leading cause of injuries in most studies [1,8,9,10,11]. Governmental policies and preventive strategies can decrease MVIs. The morbidity, mortality, and costs associated with MVIs declined after implementation of motorcycle helmet laws, seat belt laws, and measures to disallow driving under the influence [12,13,14]. Recognizing the epidemiological characteristics of pediatric injuries assists in the development of effective, preventive measures [15]. The distribution of the type of injury may change relative to different settings, areas, or times [16]. This retrospective, nationwide, population-based study investigated the epidemiology of pediatric injuries presenting to an emergency department in Taiwan.

## 2. Materials and Methods

### 2.1. Database

This nationwide population-based study was conducted using the Taiwanese National Health Insurance Research Database (NHIRD). The National Health Insurance program is a unique public health and medical insurance system that covered 99.9% of 23 million Taiwanese residents, as of the end of 2013 [17]. The Longitudinal Health Insurance Database 2000 (LHID2000) is a sub-dataset of the NHIRD consisting of data from one million people who were randomly selected from the larger NHIRD in 2000. We analyzed LHID2000 for patient data, including demographic information, medical records, prescription records, and details of medical procedures. The NHIRD is categorized according to the International Classification of Diseases, ninth revision, Clinical Modification (ICD-9-CM) system [18].

### 2.2. Study Population

We selected patients aged <18 years from the LHID2000 in 2001 as our study group and tracked them for eight years. Patients who presented to the emergency department for injuries in the following eight years were examined. The annual incidence was calculated yearly by dividing the case numbers visited emergency departments by enrolled pediatric population number. We categorized pediatric injuries into three groups according to intent: unintentional injuries (E800–E949), intentional injuries (E950–E978 and E990–E999), and undetermined injuries (E980–E989). Unintentional injuries were further categorized into 23 groups according to mechanism: railway accidents (E800–E807); MVI (E810–E819); motor vehicle non-traffic accidents (E820–E825); other road vehicle accidents (E826–E829); water transport accidents (E830–E838); air and space transport accidents (E840–E845); other vehicle accidents (E846–E848); drug poisoning (E850–E858); poisoning by other substances (E860–E869); surgical and medical adverse events (E870–E876); later complications of surgical and medical procedures (E878–E879); accidental falls (E880–E888); fires (E890–E899); accidents resulting from natural and environmental factors (E900–E909); submersion, suffocation, and foreign bodies (E910–E915); “other” factors (E916–E928); late effects of accidental injury (E929); drugs causing adverse effects in therapeutic use (E930-E949); suicide (E950–E959); homicide (E960–E969); legal intervention (E970–E978); undetermined (E980–E989); and injury resulting from operations of war (E990–E999) [19]. Some injuries may present in both intentional and unintentional injuries, such as cutting injuries; if a diagnosis of homicide or suicide was identified, these injuries would be categorized as intentional injuries (homicide or suicide).

The diagnoses were made by emergency department physicians. We collected and analyzed information on demographic data, injury type and mechanism, and epidemiological characteristics. To examine age-related epidemiology, the patients were categorized into three groups (<6 years, 6–11 years, and 12–18 years). Over the study period, etiologies with fewer than 10 patients were not subjected to further analysis.

The study was approved by the Institutional Review Board of the Tri-Service General Hospital (approval number: TSGHIRB No. B-105-06.)

### 2.3. Statistical Analysis

We used SPSS software version 19.0 (SPSS Inc., Chicago, IL, USA) for all analyses and Microsoft^®^ SQL Server^®^ 2008 software (Microsoft Unternehmen, Redmond, DC, USA) for data management. The chi-square test was used to analyze descriptive data including demographic characteristics, age, geography, level of urbanization, comorbidities, and injury types. Cox proportional hazards regression models estimated independent risk factor effects on hazard ratios with accompanying 95% confidence intervals. All models were adjusted for the covariates (gender, age, geography, urbanization, comorbidities, and injury types). A two-sided *p* < 0.05 determined statistical significance. 

## 3. Results

### 3.1. Incidences of Accidental Injuries

In total, we identified 274,028 children from the LHID2000 in 2001. Causes of pediatric injuries are displayed in Table 1. The case number and its corresponding incidence among one million children are shown. The leading causes of injuries in pediatric patients who presented to the emergency department were MVI, falls, homicide, cut injuries, poisoning, and suicide. There were no obvious seasonal variations. 

### 3.2. Temporal Trend of Accidental Injuries

**Figure 1:**
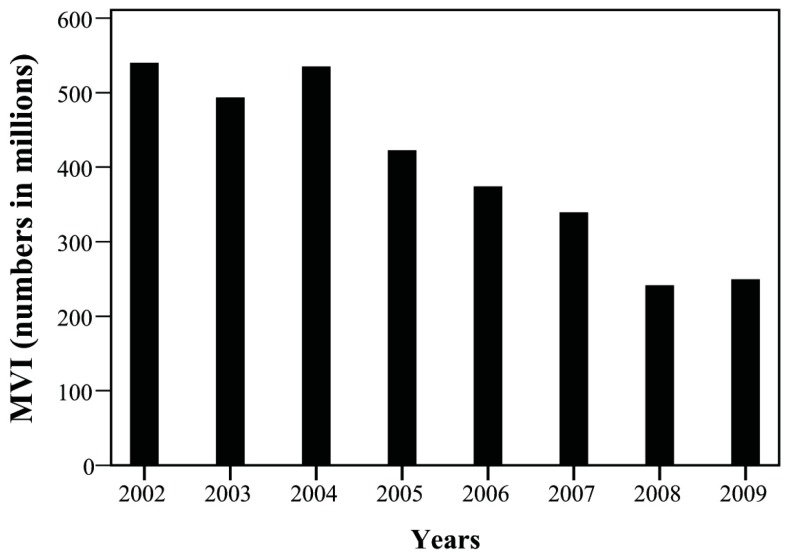
The temporal change of motor vehicle accidents between 2002 and 2009.

**Figure 2:**
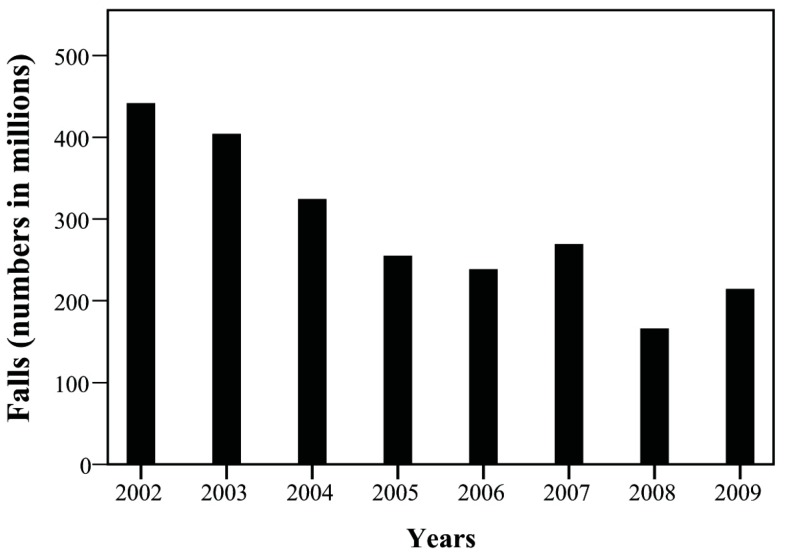
The temporal change of falls between 2002 and 2009.

**Figure 3:**
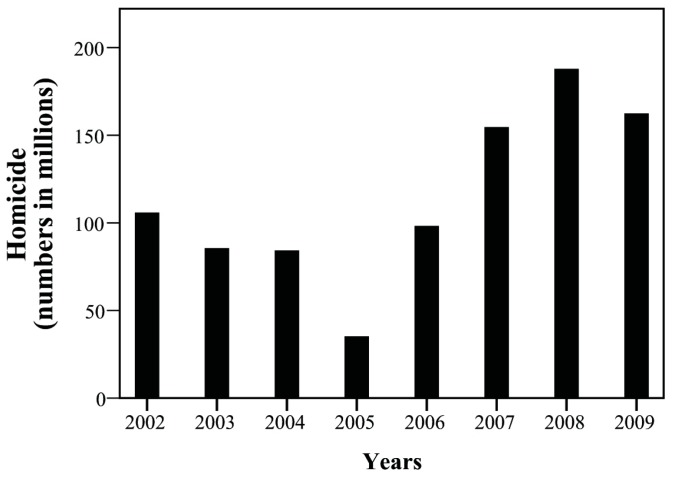
The temporal change of homicide between 2002 and 2009.

**Figure 4:**
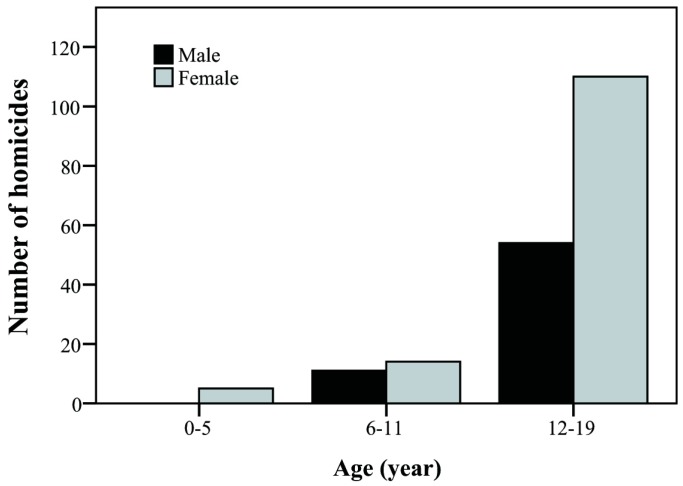
The distribution of age and gender of patients with homicide between 2002 and 2009.

The yearly trend of case number was tracked and presented as its corresponding incidence among one million children. Over the study period, overall incidence of injuries declined, particularly that of the accidents attributed to MVI and falls (Figure 1 and Figure 2). The incidence of MVI and falls was reduced by half (MVI: 540.09/1000,000→249.42/1000,000; falls: 441.56/1000,000→214.61/1000,000). We observed increased incidence of homicide in patients during this period, especially between 2007 and 2009 (Figure 3, 105.83/1000,000→62.41/1000,000). Of these, most were girls (66%) aged over 12 years (89%; Figure 4). Cox regression to evaluate the influences of independent risk factors was not performed because the detailed information of each individual was not available. Further correlation with economic conditions was explored in the discussion section. 

## 4. Discussion

The incidence of pediatric injuries declined over the course of the study, in agreement with previous studies [19,20]. As technology advances, the economy grows and motor vehicles are easily available, MVIs become a more common cause of unintentional injuries in most countries, particularly among older children. In 2008, there were 604,027 hospital-based emergency department visits at a cost of $970 million with an additional $1.8 billion required for further hospitalization [21]. The disease burden of MVIs is huge, and governmental efforts to reduce MVIs exist. Motorcycle helmet laws reduce the mortality and morbidity associated with MVI, including brain injuries [12]. Motorcycle riders wear helmets to avoid penalty. In Taiwan, motorcycle helmet laws were executed in 1997 whereupon MVI-related mortality and morbidity declined [13]. Seat belt laws were enacted in 2012. Policies that decrease alcohol use while driving reduce mortality and morbidity [22,23]. Regulations and taxes on alcohol are used in Taiwan and appear to have reduced incidence of MVIs.

A substantial decrease of falls was observed in our study (Figure 2). The Protection of Children and Youths Welfare and Rights Act of Taiwan was executed in 2003 and may contributed to the decrease of falls. According to the regulations of the act, guardians should not leave children alone in an environment that can easily cause danger or damage. Moreover, public enlightenment of safe sleep environment also contributed to the establishment of safe home environment and decrease of accidental falls [24].

**Figure 5:**
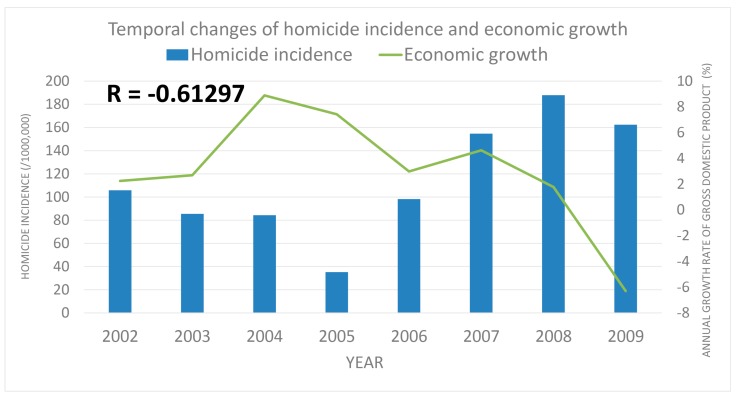
The temporal change of economic growth and homicide rate between 2002 and 2009.

Between 2002 and 2009, increased homicide rates were observed and received considerable public attention. However, between 2002 and 2009, we observed a 54% increase in the annual incidence of child homicide. The increase of homicide caught our attention. Approximately 90% were older than 12 years and two-thirds were girls. Homicide in children is a complex issue and is often related to child abuse and maltreatment [25]. There were several possible explanations for the trend of increasing child homicide, including the global financial crisis. There have been studies investigating financial crises, criminality, and homicide rates [26,27,28,29,30,31,32]. The global financial crisis between 2007 and 2009 exerted effects on public health [33,34] and affected the economy of Taiwan. We explored economic growth and gross domestic product growth during our study period using statistics of the National Bureau of Statistics, Taiwan (Table 2) [35]. Economic growth decreased to a minus gross domestic product in 2008 and 2009. The incidence of homicide increased beginning in 2006, particularly between 2007 and 2009. The financial crisis may have influenced homicide rates with the correlation coefficient −0.61297 (moderately inverse correlated, *p* = 0.041) (Figure 5). Similar correlations were also observed in previous reports, such as Greece and Korea. Greece has been significantly affected by the global financial crisis since 2008 and the unemployment rate increased from 7.6% in 2008 to 24.2% in 2012 [27]. Compared with pre-crisis period, the corresponding homicide rates and homicide-related mortality also increased (homicide rate: 1.01–1.18 vs. 1.29–1.70 per 100,000 population) [27]. A 27.6% increase of homicide-related mortality was observed in the first two years of the financial crisis [36]. Similarly, homicide mortality rates in South Korea showed a 14% increase among males and a 30% increase among females during the economic crisis in late 1990s [28]. Financial problems play an important role in homicide and the deep connection between economy and health was reinforced [26,34]. Efforts toward protection of children and youth have been made in Taiwan. For example, the Family Violence Prevention Act was implemented in 2000, and the 113 children and women protection hotline was well publicized at that time. The Protection of Children and Youths Welfare and Rights Act of Taiwan was executed in 2003. However, we found economic factors may play an important role in homicide. Reducing homicide is a multidisciplinary effort requiring concurrent support and improvements in economic, political, and social conditions. Furthermore, we also tried to find out if there was any lag between economic factors and homicides. However, the correlation between homicides and economic growth during 2006–2009 was not statistically significant (r = −0.266, *p* = 0.734). No evidence of lag between economic factors and homicides was found. Longer term data and more time data points may answer this interesting question.

Furthermore, there are differences in homicide rates between the sexes and we found girls were more susceptible to homicide in Taiwan (Figure 4). Sex differences in homicide have been reported and the predominant gender may differ in different countries, societies, races, ages, accessibility of weapons, and time. For example, victims of homicide were more common in boys in the past two decades in the United States but in girls in South Africa [37,38]. Taiwan is a patriarchal society and firearms are not allowed. Girls have less strength and may be susceptible to homicide in Taiwanese society. Detailed analyses of perpetrators, employment status, family member status, and circumstances in which homicide occurred contribute to elucidation of the reasons of gender differences. However, explicit information was unavailable for privacy and ethical concerns. Further studies are warranted to clarify the mechanisms of gender differences and the causal relationship between homicide and economic conditions.

Our study had a large study population and a broad coverage rate of 99% of Taiwanese citizens. However, there were several limitations. Detailed histories and laboratory tests were not available through the NHIRD. Therefore, further risk assessments and analyses of risk factors could not be completed. Second, patients with minor injuries may seek medical aid in outpatient clinics and were therefore not included in this study. Third, the NHIRD is organized by the ICD-9-CM. Physicians in emergency departments may miss the ICD-9-CM code if the chief complaint on admission is not accidental injury. Furthermore, full ecological time-series analyses could not be performed due to the small number of time data points. With longer term data, trends and stochastic shifts may demonstrate the association between homicide and economic growth. Detailed information regarding the perpetrators, employment status, family member status, and circumferences of homicide will contribute to clarifying the contributing factors of homicide and possible causal relationships. However, detailed data was unavailable for privacy and ethical concerns. Further studies are warranted to validate and elucidate the epidemiology and relationship between financial crises and homicide.

## 5. Conclusions

In conclusion, this nationwide population-based study provided evidence of declining pediatric injuries in emergency departments. MVIs and falls were reduced by half and new policies—including motorcycle helmet law, seatbelt laws, and increases in the alcohol tax—may contribute to continued improvement. Unfortunately, the incidence of homicide showed an increasing trend, suggesting a need for additional protections of the health and safety of children. Financial crises may contribute to increases in pediatric homicide. Multidisciplinary collaborative efforts are needed to reduce homicide and reinforce the importance of protecting children from violence.

## Figures and Tables

**Table 1:** Annual incidence of accidental injuries of pediatric emergency department.

Injury Type		Patient Number Incidence/1000,000							
	Year	2002	2003	2004	2005	2006	2007	2008	2009
Cause									
**Unintentional Injuries**		565	503	419	306	305	269	223	240
(E800–E949)		2061.83	1955.17	1765.44	1346.78	1426.52	1342.15	1196.80	1392.10
MVI *		148	127	127	96	80	68	45	43
(E810–E819)		540.09	493.65	535.11	422.52	374.17	339.28	241.51	249.42
Poisoning		5	7	5	3	5	1	4	3
(E850–E869)		18.25	27.21	21.07	13.20	23.39	4.99	21.47	17.40
Falls		121	104	77	58	51	54	31	37
(E880–E888)		441.56	404.25	324.44	255.27	238.53	269.43	166.37	214.61
Fire and Flames		4	2	0	0	1	1	1	0
(E890–E899)		14.60	7.77	0	0	4.68	4.99	5.37	0
Drowning		1	1	0	2	0	1	1	1
(E910)		3.65	3.89	0	8.80	0	4.99	5.37	5.80
Suffocation		0	1	1	0	1	0	0	0
(E911–E913)		0	3.89	4.21	0	4.68	0	0	0
Cut Injuries		25	18	10	4	8	5	14	11
(E920)		91.23	69.97	42.13	17.6	37.42	24.95	75.14	63.80
Other		59	61	33	20	21	14	7	12
(E916–E928)		215.31	237.11	139.04	88.02	98.22	69.85	37.57	69.60
**Intentional Injuries**		73	43	49	38	48	63	60	50
(E950–E999)		266.40	167.14	206.46	167.25	224.50	314.33	322.01	290.02
Homicide		29	22	20	8	21	31	35	28
(E950-E959)		105.83	85.51	84.27	35.21	98.22	154.67	187.84	162.41
Suicide		3	2	4	3	5	7	3	3
(E960–E969)		10.95	7.77	16.85	13.20	23.39	34.93	16.10	17.40
Other		41	19	25	27	22	25	22	19
(E980–E989)		149.62	73.85	105.34	118.83	102.90	124.73	118.07	110.21

Injuries with fewer than 10 patients are not shown in the table, MVI *: motor vehicle accidents.

**Table 2:** The economic growth and gross domestic product in Taiwan, 2002–2009.

Year	Economic Growth (%)	Per Capita Gross Domestic Product		Per Capita National Income	
		Value (NTD *)	Annual Growth Rate (%)	Value (NTD *)	Annual Growth Rate (%)
2002	5.57	13,750	2.25	12,270	2.8
2003	4.12	14,120	2.69	12,642	3.03
2004	6.51	15,388	8.89	13,735	8.65
2005	5.42	16,532	7.43	14,602	6.31
2006	5.62	17,026	2.99	14,974	2.55
2007	6.52	17,814	4.63	15,401	2.85
2008	0.7	18,131	1.78	15,388	−0.08
2009	−1.57	16,988	−6.3	14,398	−6.43

NTD *: New Taiwan Dollars; 1 US Dollar = 32 NTD.
